# Immunologic Strategies in Pancreatic Cancer: Making *Cold* Tumors *Hot*

**DOI:** 10.1200/JCO.21.02616

**Published:** 2022-07-15

**Authors:** Nicholas A. Ullman, Paul R. Burchard, Richard F. Dunne, David C. Linehan

**Affiliations:** ^1^ University of Rochester Medical Center, Rochester, NY

## Abstract

The rising incidence and persistent dismal 5-year overall survival of pancreatic ductal adenocarcinoma (PDAC) highlight the need for new effective systemic therapies. Immunotherapy has shown significant benefits in solid organ tumors, but has thus far been disappointing in the treatment of PDAC. There have been several promising preclinical studies, but translation into the clinic has proved to be challenging. This is likely a result of PDAC's complex immunosuppressive tumor microenvironment that acts to insulate the tumor against an effective cytotoxic immune response. Here, we summarize the mechanisms of immunosuppression within the PDAC tumor microenvironment and provide an up-to-date review of completed and ongoing clinical trials using various immunotherapy strategies.

## INTRODUCTION

Pancreatic ductal adenocarcinoma (PDAC) is projected to become the second leading cause of cancer-related mortality by 2030.^1^ Despite modest advances in conventional systemic therapies, the 5-year overall survival (OS) for PDAC remains a dismal 11%,^2^ in part because of its advanced stage at presentation precluding curative-intent resection and a high propensity for recurrence. Traditional fluorouracil- or gemcitabine-based chemotherapies, with or without radiation, are standard of care for patients with unresectable disease;^3^ however, development of more effective systemic therapies remains a significant unmet clinical need.

**KEY POINTS**

- Pancreatic adenocarcinoma possesses several intrinsic and extrinsic properties that insulate malignant cells from an effective adaptive immune response.
- Thus far, no single immunotherapy strategy has proved to be effective, warranting investigation of combination approaches to improve efficacy.
- Ongoing clinical trials evaluating combination immunotherapy strategies will demonstrate the role of immunotherapy in the treatment of pancreatic adenocarcinoma.

**CONTEXT**

**Key Objective**
What are the current strategies being investigated to overcome the profoundly immunosuppressive pancreatic adenocarcinoma (PDAC) tumor microenvironment (TME)?
**Knowledge Generated**
PDAC uses several intrinsic and extrinsic mechanisms to develop an immunosuppressive TME, thus rending previous immunotherapy strategies ineffective. Combination immunotherapy strategies targeting these mechanisms are currently being investigated.
**Relevance**
Overcoming the immunosuppressive TME will allow for immunotherapy to become a valuable treatment option in PDAC. Well-designed clinical trials with robust correlative science are necessary to further understand potential mechanisms of immune evasion and inform future studies.

Advances in immunotherapies, specifically immune checkpoint blockade (ICB), have improved treatment options for some historically chemotherapy-refractory malignancies. In the past 10 years, ICB has shown efficacy in metastatic melanoma, renal cell carcinoma, colorectal cancers with microsatellite instability, non–small-cell lung cancer, Hodgkin's lymphoma, and various other cancers.^4-7^ Anti–programmed death-1 (anti–PD-1) with or without anti–cytotoxic T-cell lymphocyte-4 therapy is now the standard of care for patients with advanced melanoma.^8^

Despite the successes of ICB, PDAC has been largely refractory to ICB monotherapy.^9^ Studies of single-agent ICB and dual-agent ICB with anti–PD-1 and anti–cytotoxic T-cell lymphocyte-4 antibodies have resulted in overall response rates (ORRs) of 0%^10-12^ and 3%, respectively.^12^ These disappointing results, contrasted with the marked effectiveness of ICB in other solid tumors, have influenced a body of research to identify and harness immunologic pathways that could be key to unlocking immunotherapy as a viable treatment option for the typically immunologically cold pancreatic cancer. Here, we summarize the mechanisms of immunosuppression within the PDAC tumor microenvironment (TME) and provide an up-to-date review of promising immunotherapy strategies.

## PDAC-INTRINSIC PROPERTIES LEADING TO IMMUNE EVASION

**FIG 1.**
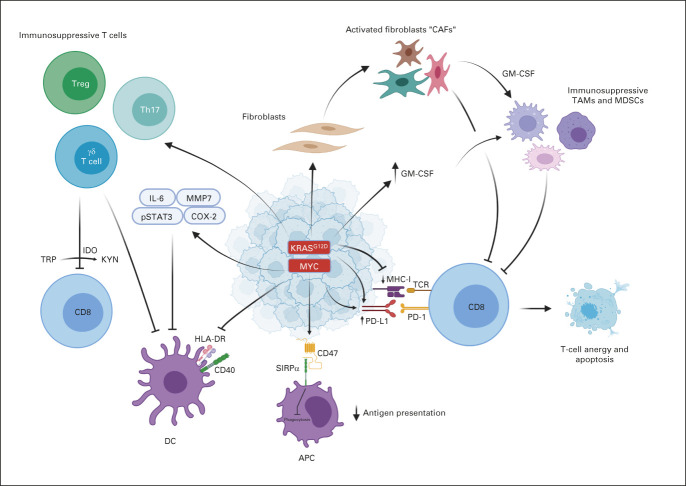
PDAC-intrinsic immunoevasive properties. APC, antigen-presenting cell; CAF, cancer-associated fibroblasts; DC, dendritic cell; GM-CSF, granulocyte-macrophage colony-stimulating factor; HLA-DR, human leukocyte antigen-DR isotype; IDO, indoleamine 2,3-dioxygenase; IL, interleukin; KYN, kynurenine; MDSCs, myeloid-derived suppressor cells; MHC-1, major histocompatibility complex-1; PD-1, programmed death-1; PDAC, pancreatic adenocarcinoma; PD-L1, programmed death ligand-1; SIRPα, signal regulatory protein alpha; TAM, tumor-associated macrophages; TCR, T-cell receptor; Treg, regulatory T-cell; TRP, tryptophan.

PDAC possesses several intrinsic properties that result in evasion of an effective immune response (Fig 1). In general, tumor-specific antigens (TSAs) are expressed only on malignant cells, thus providing excellent specificity for antitumor T-cell cytotoxicity, with antigen strength correlating with the level of antitumor immune response.^13-17^ Retrospective data of surgically resected specimens suggest a survival advantage in the minority of patients whose tumors exhibit high levels of both TSAs and CD8+ T-cell infiltrate.^18^ Despite this association, CD8+ T cells demonstrate decreased interferon-gamma and other activation markers, indicating other immunosuppressive factors at play.^19^

PDAC oncogenes and their downstream effects contribute to the immunosuppressive TME. Mutated KRAS, resulting in constitutive activation, is found in 92% of pancreatic cancer^20^ and is associated with several downstream effects including production of granulocyte-macrophage colony-stimulating factor (GM-CSF), leading to recruitment of immunosuppressive myeloid cells^21^; promotion, formation, and maintenance of the fibroinflammatory stroma^22^; upregulation of programmed death ligand-1 (PD-L1) expression through mRNA stabilization^23^; increased CD73 expression leading to elevated immunosuppressive extracellular adenosine^24^; downregulation of major histocompatibility complex-1 and increasing regulatory T cells (Tregs)^25^; and induction of immunosuppressive Th17 and gamma-delta T cells.^26^

In addition to immunosuppressive oncogenes, PDAC cells possess variable mechanisms that impair antigen presentation and cytotoxic lymphocyte (CTL) function. PDAC cells selectively target major histocompatibility complex-1 molecules for lysosomal degradation through an autophagy-dependent mechanism.^27^ Preclinical inhibition of autophagy with hydroxychloroquine resulted in decreased tumor growth^28^ and synergized with dual ICB to enhance antitumor immune response.^27^ In addition, PDAC cells contain a high proportion of CD47 that prevents phagocytosis and antigen presentation by antigen-presenting cells (APCs).^29^ Anti-CD47 antibody-mediated phagocytosis of cancer cells by macrophages results in increased priming of CD8+ T cells and reduced immunosuppressive Tregs.^30^ PDAC cells also produce indoleamine 2,3-dioxygenase (IDO) to catalyze the degradation of tryptophan, a necessary component of cytotoxic T-cell survival and activation, thereby inducing T-cell apoptosis and anergy.^31^ Furthermore, PDAC cells downregulate the expression of human leukocyte antigen-DR isotype and CD40, resulting in immature dendritic cells (DCs) capable of directly suppressing effector CD8+ T cells.^32^ Overall, PDAC's intrinsic immunosuppressive properties afford several mechanisms to subvert the normal host immune response, posing unique challenges to immunotherapeutic drug development in this tumor type.

## THE IMMUNOSUPPRESSIVE PDAC MICROENVIRONMENT

### Stromal Components—Cancer-Associated Fibroblasts and the Desmoplastic Reaction

**FIG 2.**
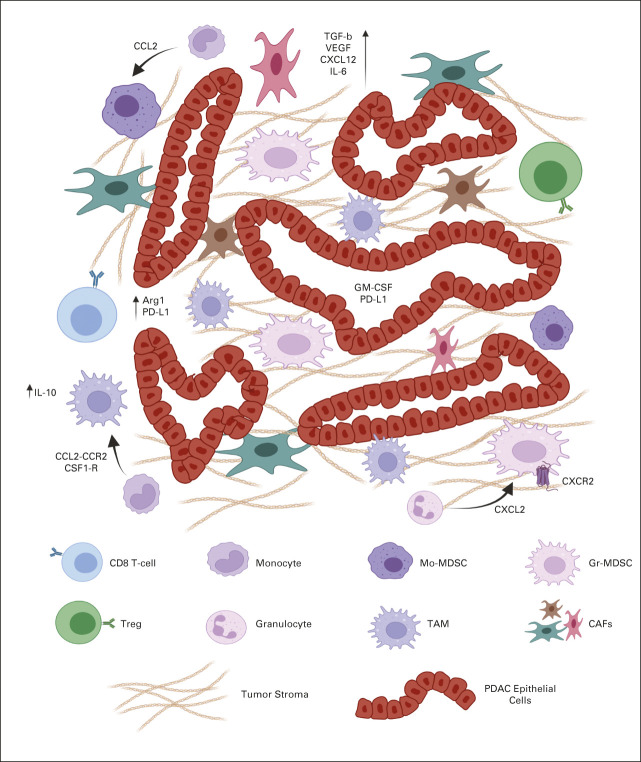
The highly immunosuppressive tumor microenvironment of pancreatic ductal adenocarcinoma. Pancreatic tumor cells, myeloid cells (Mo-MDSCs, TAMs, and Gr-MDSCs), and fibroblasts within the tumor microenvironment interact through various ligands, cytokines, and chemokines that disrupt antitumor immunity.^48^ CAF, cancer-associated fibroblasts; GM-CSF, granulocyte-macrophage colony-stimulating factor; Gr-MDSC, granulocytic MDSC; IL, interleukin; MDSC, myeloid-derived suppressor cell; Mo-MDSC, monocytic MDSC; PD-L1, programmed death ligand-1; PDAC, pancreatic ductal adenocarcinoma; TAM, tumor-associated macrophage; TGF, transforming growth factor; Treg, regulatory T-cell; VEGF, vascular endothelial growth factor.

Although PDAC cells have intrinsic properties leading to immune evasion, their interaction with the surrounding TME poses a larger, more complex barrier to effective immunotherapy strategies (Fig 2). The histologic hallmark of PDAC is a heavily desmoplastic microenvironment that accounts for approximately 70% of tumor tissue, with increased fibrosis shown to be an independent prognostic factor.^33,34^ Pancreatic stellate cells (PSCs; activated PSCs have been referred to as cancer-associated fibroblasts [CAFs]) produce this fibrotic environment and exhibit several factors that promote tumorigenesis and abrogate antitumor immunity.^35^

The marked desmoplasia results in elevated interstitial fluid pressure limiting perfusion and diffusion of small molecule therapies secondary to intratumoral small vessel collapse.^36^ The associated hypoperfusion produces an overall hypoxic environment resulting in a Treg-mediated CD8+ T-cell inhibition.^37^ Preclinical work targeting hyaluronic acid (HA) through enzymatic degradation resulted in normalization of interstitial fluid pressure and permanent remodeling of the TME, leading to doubled OS when paired with chemotherapy.^36,38^

Beyond the physical barrier, CAFs appear to limit the migration of CTLs to the juxtatumoral stromal compartments through hyperactivation of focal adhesion kinase (FAK) and overproduction of C-X-C Motif Chemokine Ligand 12 (CXCL12), a ligand of C-X-C Motif Chemokine Receptor 4 (CXCR4), overall inhibiting T-cell priming.^39^ Preclinical models of FAK inhibition limited tumor progression, doubled survival, decreased immunosuppressive cells, and synergized with ICB therapy.^40^ In addition, the use of a CXCR4 antagonist increased CD8+ T-cell accumulation and acted synergistically with anti–PD-L1 antibody to decrease tumor burden in preclinical models.^41^ CAFs are capable of diminishing CTL function through secretion of soluble substances such as interleukin-10 (IL-10), transforming growth factor-β, vascular endothelial growth factor, prostaglandin E1, IDO, arginase, and expression of PD-L1.^40^

In addition to their interaction with CTLs, CAFs interact with immunosuppressive myeloid cells through secretion of inflammatory cytokines such GM-CSF, IL-6, vascular endothelial growth factor, and macrophage colony-stimulating factor. These pathways have been shown to encourage peripheral blood mononuclear cell differentiation toward immunosuppressive myeloid-derived suppressor cells (MDSCs),^42^ whereas CAF-derived GM-CSF directly leads to tumor cell proliferation, invasion, and transendothelial migration.^43^ In turn, myeloid cell–derived IL-1β can reprogram normal fibroblasts into proinflammatory CAFs that further mediate tumor-enhancing inflammation by recruiting and polarizing macrophages toward a cancer-promoting M2 phenotype.^44^ This complex interaction between tumor cells, CAFs, T cells, and myeloid cells underscores the intertwined protumor mechanisms within the various components of the TME.

### Cellular Components

#### Myeloid cells.

The PDAC TME is characterized by a robust immune infiltrate, which comprises nearly 50% of its cellular component and is largely composed of CD45+ bone marrow–derived immune cells.^16,22,45^ PDAC induces an altered state of myelopoiesis, recruitment, and repolarization of these cells to promote their accumulation and immunosuppressive properties within the TME.^46,47^ These intratumoral MDSCs are composed of myeloid progenitors and immature mononuclear cells, referred to as granulocytic MDSC (Gr-MDSCs) and monocytic MDSC, respectively. Tumor-associated macrophages (TAMs), in contrast to MDSCs, are mature cells derived from either the bone marrow or resident tissue macrophages.^48,49^ Elevated peripheral and intratumoral levels of inflammatory myeloid cells have been associated with poor clinical outcomes.^50-52^

TAMs are dominated by an M2 phenotype, virtually eliminating an M1 (antitumor phenotype) response. M2 TAMs produce IL-10 that maintains functional Treg populations and drive the development of Th2 cells, which secrete IL-4 and potentiate the development of additional TAMs.^51^ Inhibiting IL-10 resulted in increased IL-12 secretion from DCs and led to improved CTL infiltration and response to chemotherapy.^53^ TAMs can also directly induce T-cell apoptosis through their expression of PD-L1^54^ and Dectin-1/galectin-9 axis^55^ and inhibit CTLs through production of arginase-1–depriving cytotoxic effector T cells of L-arginine, a key nutrient to support viability and expansion.^51^

Similar to TAMs, MDSCs deplete micronutrients through arginase-1–dependent consumption and L-cysteine sequestration to downregulate the T-cell receptor complex (TCR) and cause proliferative arrest of antigen-activated T cells.^47^ Furthermore, MDSCs are potent generators of reactive oxygen and nitrogen species that impair TCR activity and interfere with IL-2, a potent proinflammatory cytokine.^51^ In addition to TCR disruption, MDSCs have the ability to cause T-cell apoptosis, inhibit natural killer cells, and increase the activation and expansion of Tregs. Genetic ablation of CXCR2, a chemokine receptor found predominantly on Gr-MDSCs, led to increased T-cell infiltration into the tumor stroma.^56^ In an orthotopic model, inhibition of MDSCs via CXCR2 blockade led to decreased MDSCs within the TME, decreased fibrosis, and acted synergistically with ICB.^57^

Preclinical studies have identified a potential mechanism of resistance to TAM-targeted therapy by a compensatory increase in CXCR2+ Gr-MDSCs; dual inhibition of both TAMs and Gr-MDSCs demonstrated increased survival.^58^ Modulation of the myeloid receptor CD11b reduced intratumoral TAMs and MDSCs, repolarized M2 TAMs to an antitumor M1 phenotype, and increased infiltration of activated CD8+ T cells in preclinical models. When combined with anti–PD-1 antibody or chemotherapy, these immunomodulatory effects translated into potent antitumor effects and prolonged survival in orthotopic PDAC murine models.^59^ It is evident through a variety of mechanisms that PDAC co-opts myeloid cell pathways to render a cytotoxic T-cell response ineffective and thus requires consideration when developing immunotherapy strategies for this disease.

#### Dendritic cells.

Conventional dendritic cells (cDCs) are professional APCs adept at presenting exogenous and/or endogenous antigens to T cells. Recruitment, retention, and spatial positioning of cDCs within the TME are limited by PDAC-derived proinflammatory cytokines and resulting immunosuppressive myeloid infiltrate.^60^ Reduced cDC concentrations appear to be influenced by high levels of cyclooxygenase 1 and 2 and decreased levels of locally available cDC growth factors such as the natural killer cell–producing fms-like tyrosine kinase 3 ligand (FLT3L).^61^

Soluble inhibitory factors not only work to exclude cDCs but also to limit their function as APCs. TAM- and Treg-generated IL-10 suppresses cDC production of IL-12, a costimulatory molecule necessary to mount an adaptive immune response.^32^ cDCs are also subject to increasing apoptosis secondary to increased levels of IL-6.^60^ Combination therapy with a CD40 agonist (a stimulatory ligand for T-cell activation) and FLT3L restored cDC abundance, improved tumor infiltration, and resulted in superior control of tumor outgrowth in a preclinical model.^61^

#### B cells.

Recent studies have linked B cells to PDAC as resected human PDAC exhibited increased CD20 and Ig expression relative to normal pancreata,^62^ whereas depletion of B cells with anti-CD20 monoclonal antibodies inhibited progression of pancreatic intraepithelial neoplasia preclinically.^63^

#### T cells.

Although a relative minor component of the PDAC immune infiltrate, the T-cell infiltrate exhibits both anti- and protumor immunologic effects and includes effector CD8+, CD4+ (both Th1 and Th2 helper cells), FoxP3+ Tregs, Th17+, and γδ T cells. There is a relative paucity of cytotoxic effector CD8+ T cells within the TME, comprising < 7% of the total leukocyte infiltrate.^64^ In addition to their limited presence, these effector cells are often functionally deficient as they express various coinhibitory molecules.^64^

CD4+ helper T cells are found with greater frequency within the TME relative to CD8+ T cells and display a tumor-promoting Th2 phenotype.^65^ Although less frequent than Th2 cells, Treg density increases with disease progression and has been found to correlate with lymph node metastases and poor survival.^66,67^ PDAC cells produce a host of cytokines that are associated with Treg migration and accumulation including CCL5,^68^ transforming growth factor-β, and IL-10.^69^ These immunosuppressive T cells possess several protumor immunologic effects including restraint of tumor-associated DC expansion and suppression of the costimulatory ligands CD86 and CD40, which are necessary for CD8+ T-cell activation^32^ and promotion of local immune suppression.^70,71^ Eliminating Tregs in a preclinical model allowed DCs to induce a potent antitumor immune response that was CD8+-dependent.^32^ However, Treg depletion in a spontaneous murine model did not affect CD8 T-cell recruitment, suggesting that Treg elimination alone is insufficient to restore productive T-cell immunity.^72^

The complex T-cell populations, composed of both pro- and antitumor cells, require selective stimulatory and inhibitory strategies to elicit an effective adaptive immune response. Although an effective CD8+ T-cell response is the final pathway of most immunotherapy regimens, PDAC possesses several mechanisms to subvert activation of adaptive immunity through ICB alone. Combination therapies are likely required to garner an effective immunotherapy regimen.

## CURRENT STRATEGIES FOR IMMUNOTHERAPY

### Stromal Targeting

Strategies disrupting components of the desmoplastic PDAC stroma have been met with variable results. Despite preclinical success of enzymatic degradation of HA using pegvorhyaluronidase (PEGPH20), its addition to gemcitabine/nab-paclitaxel was evaluated in the phase III HALO 109-301 trial of patients with HA-high PDAC and demonstrated a slight increase in ORR, 47% versus 36% (ORR ratio, 1.29 [95% CI, 1.03 to 1.63]), but no change in OS (hazard ratio [HR], 1.00; 95% CI, 0.80 to 1.27; *P* = .97) or progression-free survival (PFS; HR, 0.97; 95% CI, 0.75 to 1.26).^73^ These disappointing results of HA-targeted therapy have led to pairing PEGPH2O with other immunotherapies, and investigations of combining PEGPH20 with ICB are ongoing (Table 1).

**TABLE 1.**
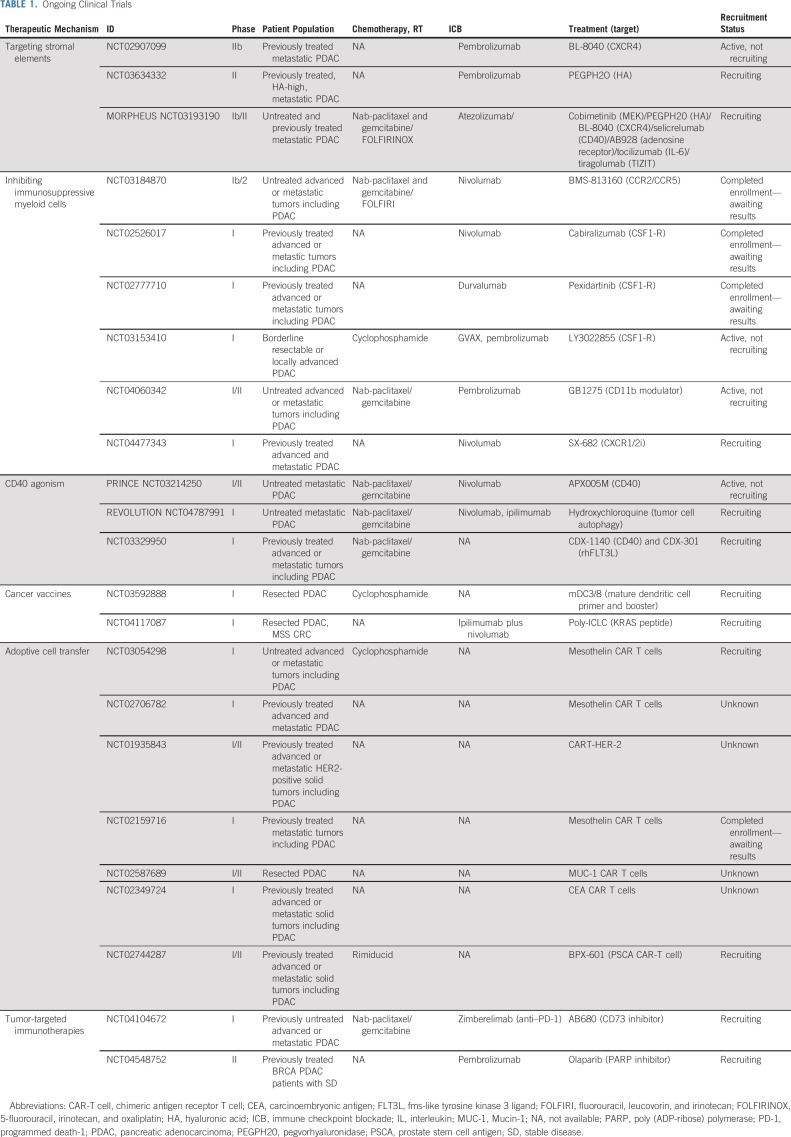
Ongoing Clinical Trials

Another recent stromal-associated strategy includes CXCR4/CXCL12 axis disruption. The phase IIa COMBAT trial evaluated BL-8040, a CXCR4 inhibitor, in combination with anti–PD-1 therapy with or without chemotherapy in previously treated patients with metastatic PDAC. Treatment with BL-8040 resulted in decreased suppressive cell types within the TME and promotion of T-cell infiltration. The cohort treated with BL-8040 plus ICB and chemotherapy demonstrated encouraging clinical outcomes with an ORR of 32% and a disease control rate (DCR) of 77%.^74^

Contrasting the encouraging results of the COMBAT trial, other studies evaluating CXCR4 inhibition have resulted in poorer treatment responses. A best overall response of stable disease (SD) in three of eight patients was found with combination treatment of LY2510924 (a CXCR4 peptide antagonist) and anti–PD-L1 in patients with advanced refractory PDAC, similar to responses seen with ICB alone.^75^

Two other stromal-targeting strategies include inhibition of FAK and the upstream Janus kinase–signal transducers and activators of transcription (JAK-STAT) signaling pathway. A phase I trial pairing defactinib, a small molecule inhibitor of FAK, and anti–PD-1 therapy with gemcitabine showed modest results with 2 of 27 and 14 of 27 patients with PDAC showing partial response (PR) or SD, respectively. Paired biopsies demonstrated increased CD8+ T-cell infiltration and proliferation, whereas Tregs, macrophages, and stromal density decreased with treatment.^76^ The JAK-STAT pathway plays a key role in activation of PSCs.^77^ Unfortunately, the JANUS1 and JANUS 2 trials combining ruxolitinib and capecitabine showed no difference in OS (JANUS 1: HR, 0.969; 95% CI, 0.74 to 1.2; JANUS 2 HR, 1.58; 95% CI, 0.89 to 2.83) or PFS (JANUS I: HR, 1.06; 95% CI, 0.82 to 1.35; JANUS 2: HR, 1.17; 95% CI, 0.69 to 1.98).^78^

The ongoing phase Ib/II Morpheus trial in metastatic PDAC seeks to combine several immunotherapies in a variety of treatment settings. The trial is enrolling both pretreated and treatment-naïve patients with metastatic PDAC and randomly assigns them to a variety of treatment arms including pairing atezolizumab (anti–PD-L1) with PEGPH20 or BL-8040 as second-line treatment. Of note, one arm of this trial that combined atezolizumab with cobimetinib (a MEK inhibitor) in 14 patients with refractory PDAC showed no objective responses.^79^ Although translation of stromal-targeting strategies has thus far been met with challenges, correlative studies have been insightful. Further results are pending from the Morpheus trial, which will shed light on combining stromal- and immune cell–targeting therapies.

### Myeloid Suppression/Reprogramming

The CCL2-CCR2 chemokine axis, which plays a role in recruiting TAMs into the TME, has been a molecular pathway targeted by investigators. In a phase I study of locally advanced PDAC, the combination of PF-04136309 (an oral CCR2 inhibitor) and 5-fluorouracil, irinotecan, and oxaliplatin (FOLFIRINOX) resulted in a 49% ORR and a 97% DCR.^80^ However, an additional phase I/II study pairing the same oral CCR2 inhibitor with gemcitabine/nab-paclitaxel was terminated early because of lack of efficacy.^81^ A combinatory CCR2/CCR5 inhibitor with or without chemotherapy and anti–PD-1 therapy trial in metastatic colorectal and PDAC has finished enrollment with awaiting results (ClinicalTrials.gov identifier: NCT03184870).

Similar to CCR2 inhibition, CSF1-R inhibition leads to disruption of TAM recruitment and repolarization to promote antigen presentation, thus increasing T-cell activation through synergizing with ICB.^82^ Unfortunately, a randomized phase II study of cabiralizumab (anti–CSF-1R) + anti–PD-1 therapy with or without chemotherapy in advanced PDAC did not meet its primary end point of increasing PFS (ClinicalTrials.gov identifier: NCT03336216). However, several studies using CSF1-R inhibitors with various combinations of chemotherapy and immunotherapy are ongoing (Table 1).

Contrasting TAM-targeted therapy, a phase I study evaluating SX-682, a CXCR2 inhibitor targeting Gr-MDSCs, in combination with anti-PD1 therapy as maintenance therapy in patients with stable unresectable PDAC after first-line chemotherapy is currently ongoing (ClinicalTrials.gov identifier: NCT04477343). In addition, dual inhibition of both TAM and G-MDSC populations through CD11b modulation in combination with anti–PD-1 therapy and chemotherapy is currently being explored with minimal adverse effects reported.^83^ With many ongoing studies, myeloid cell targeting represents a promising component of immunotherapy strategies to mitigate the potent immunosuppressive TME.

### B-Cell Targeting

B cells were recently implicated as contributors to the PDAC-immunosuppressive TME and were targeted in a phase III trial evaluating ibrutinib, a Bruton's tyrosine kinase inhibitor, in combination with chemotherapy. Ibrutinib, used in the treatment of various hematologic malignancies, had demonstrated reduced stromal fibrosis and decreased tumor progression in preclinical PDAC models.^84^ However, the phase III RESOLVE study examining treatment-naïve patients with metastatic PDAC found that the combination of gemcitabine/nab-paclitaxel with ibrutinib resulted in no improvement in median OS (9.7 *v* 10.8 months; *P* = .3225) and reduced PFS (5.3 *v* 6.0 months; *P* < .0001) and ORR (29% *v* 42%; *P* = .0058) when compared with standard chemotherapy.^85^

## INCREASED T-CELL ACTIVATION BEYOND ICB

### CD40 Agonist

In addition to targeting the immunosuppressive components of the TME, a complementary strategy is to enhance the cytotoxic capabilities of the adaptive immune system. CD8+ T cells express both coinhibitory and costimulatory receptors, and activating the latter may be able to compensate for the intrinsic and environmentally poor antigen quality and presentation. Agonistic antibodies to these costimulatory receptors, namely, anti-CD40, have shown promise in PDAC.^86^

Correlative work from phase I studies of isolated CD40 agonism demonstrated CD8+ T-cell enrichment, increased mature DCs, reduced M2 TAMs, and increased B-cell expression of costimulatory molecules.^87,88^ The increasing T-cell response seen with CD40 agonists was associated with increased expression of PD-L1 within the PDAC TME, suggesting that pairing ICB with CD40 agonists may be a valuable strategy.^89^ The phase Ib PRINCE trial combining gemcitabine/nab-paclitaxel and the CD40 agonist APX005M with or without anti-PD1 antibody in untreated metastatic PDAC demonstrated an overall 58% response rate among all treated patients, while showing a tolerable safety profile.^90^ In the phase II PRINCE trial, chemotherapy plus anti-PD1 antibody and APX005M did not show an improvement in OS when compared with historical controls (*P* = .236). Interestingly, chemotherapy combined with either anti-PD1 antibody or APX005M resulted in improved 1-year OS when compared with historical controls (57% [*P* = .007] and 51% [*P* = .029], respectively *v* 35% in historical controls).^91^ Correlative studies are ongoing to identify potential biomarkers and resistant mechanisms of therapies. Building on the findings of the PRINCE trial, the Revolution Platform study (ClinicalTrials.gov identifier: NCT04787991) will combine gemcitabine/nab-paclitaxel with nivolumab plus ipilimumab or hydroxychloroquine plus ipilimumab as first-line treatment for metastatic pancreatic adenocarcinoma. This trial is currently enrolling.

### PDAC Vaccines

Similar to other immunotherapy strategies for PDAC, vaccination has been met with varying success. GVAX, an irradiated allogeneic whole-tumor cell vaccine in which PDAC cells are engineered to express GM-CSF, induces T-cell infiltration when administered before resection.^92^ In patients with previously treated PDAC, a phase II study of cyclophosphamide and GVAX with or without CRS-207, a bacterium-based vaccine, found that those receiving CRS-207 experienced improved OS when compared with second-line chemotherapy (6.1 months *v* 3.9 months [HR], 0.59; *P* = .02).^93^ Although this study appeared to enhance CD8+ T-cell response, the larger Phase IIb ECLIPSE study examining the combination of cyclophosphamide/GVAX/CRS-207 failed to show a difference in OS compared with single-agent chemotherapy (*P* = not significant; HR, 1.17; 95% CI, 0.84 to 1.64).^94^ The addition of anti–PD-1 therapy to GVAX/cyclophosphamide/CRS-207 yielded an OS and a PFS of 5.88 months and 2.23 months, respectively, not significantly different from GVAX/cyclophosphamide/CRS-207 alone.^95^

Vaccine therapy has been deployed in the adjuvant setting with mixed results. Algenpantucel-L, a whole-cell vaccine genetically engineered to facilitate complement and antibody-dependent cytotoxicity, was added to adjuvant standard-of-care chemotherapy in a phase II study. This single-arm study demonstrated favorable results finding the 1-year disease-free survival (DFS) and OS to be 62% and 86%, respectively.^96^ Unfortunately, the randomized phase III IMPRESS study examining this approach failed to demonstrate a survival advantage compared with controls (ClinicalTrials.gov identifier: NCT01072981). Algenpantucel-L was also evaluated in borderline resectable disease, but again did not improve median OS (HR, 1.02; 95% CI, 0.66 to 1.58; *P* = .98) nor PFS (HR, 1.33; 95% CI, 0.72 to 1.78; *P* = .59) when compared with standard therapy.^97^

Another targetable antigen for vaccine therapy is Mucin-1 (MUC-1). MUC-1 is a transmembrane protein involved in oncogenic signaling to increase invasion, angiogenesis, and metastasis.^98^ A phase I study of resected PDAC using MUC-1 peptide has shown that mucin vaccination increased intratumoral and peripheral blood CD8+ T cells, with low but detectable mucin-specific T-cell response.^99^

As mentioned above, mutated KRAS is found in more than 92% of PDAC, presenting itself as an ideal vaccination target. Early studies paired mutant KRAS vaccine with the GM-CSF peptide in the adjuvant setting, and although it was found to be safe, an immune response was seen in only 11% of patients as measured by delayed type hypersensitivity.^100^ This was in contrast to the results of a phase I/II trial evaluating a mutant KRAS peptide vaccine in an adjuvant setting that demonstrated an immune response in 85% of patients. In this study, 10-year survival was found in 20% of immune responders versus 0% in matched controls.^101^ Adjuvant trials using mutant KRAS–specific DC vaccination alone or with dual ICB are currently ongoing (ClinicalTrials.gov identifiers: NCT03592888 and NCT04117087).

PDAC vaccines have demonstrated ability to enhance an antitumor T-cell response; however, the number of nonresponders is not insignificant, suggesting that vaccination therapy is insufficient as monotherapy and that additional mechanisms of immune evasion are present.

### Adoptive Cell Transfer (chimeric antigen receptor T cell)

Rapid advances in the field of adoptive cell transfer have resulted in unprecedented clinical outcomes for patients with hematologic malignancies^102^; however, these promising results have not translated to PDAC. Adoptive cell transfer refers to harvesting and ex vivo expansion of the patient's own tumor antigen–specific T cells. Enhanced T cells are then reinfused to produce a robust adaptive immune response. Of the adoptive cell transfer therapies, chimeric antigen receptor T-cell (CAR-T) therapy is the most clinically developed.

An early trial of CAR T cells in unresectable or recurrent PDAC used MUC-1 peptide-pulsed DCs and activated T lymphocytes.^103^ Of 20 treated patients, one patient with multiple lung metastases experienced complete response (CR) and five had SD. Unfortunately, several subsequent studies have attempted to use CAR-T technology in PDAC, with the majority lacking efficacy (Table 2).

**TABLE 2.**
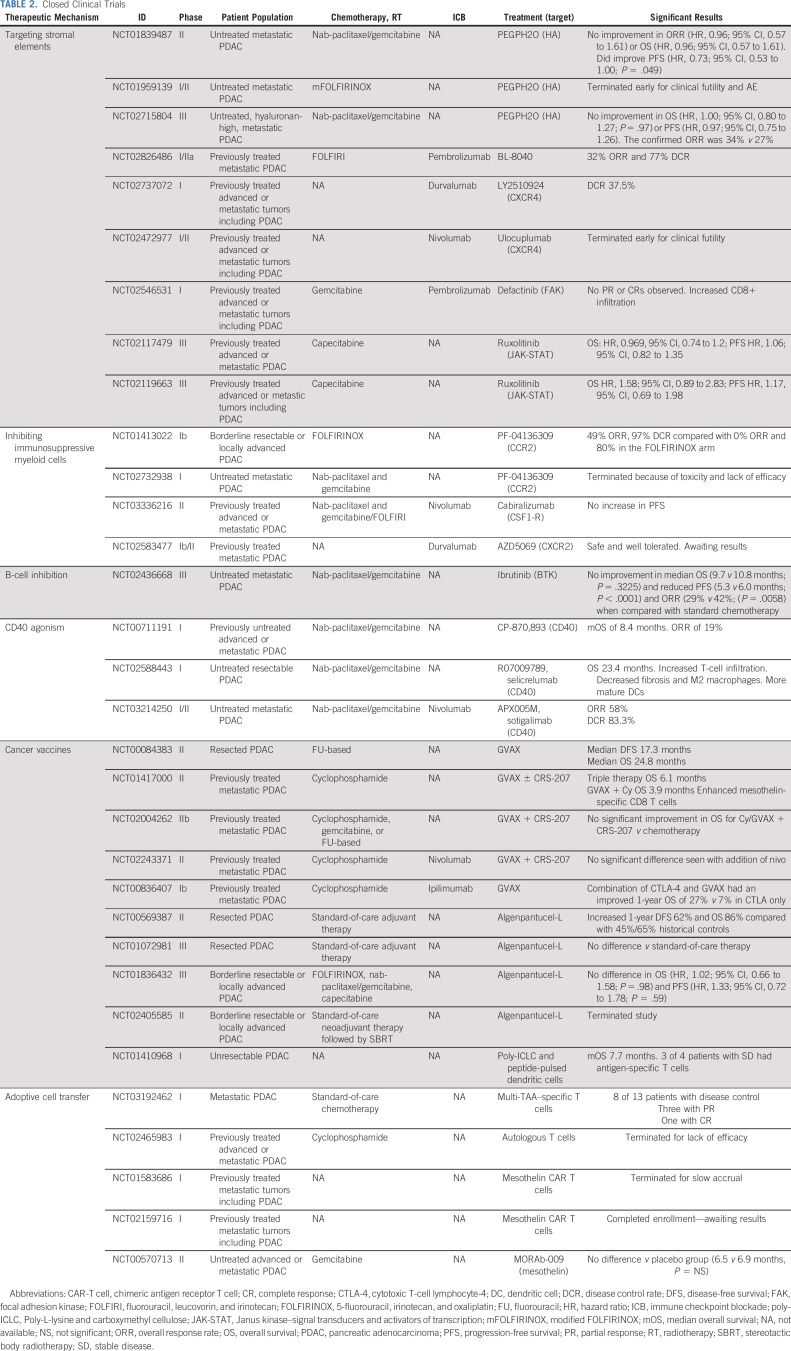
Closed Clinical Trials

In the adjuvant setting, MUC-1–primed CAR-T in combination with gemcitabine demonstrated a DFS of 15.8 months and an OS of 24.7 months. Long-term DFS in this study was independently associated with the average number of CTLs administered (*P* = .0133).^104^ A recent phase I study of patients with metastatic PDAC treated with a combination of standard-of-care chemotherapy and CAR-T demonstrated a DCR in 8 of 13 patients, an increase compared with historical controls. Of these eight metastatic patients, three had PR and one had CR.^105^

Although adoptive cell transfer is a new and promising field of immunotherapy, it possesses many limitations. Antigen selection poses a significant hurdle for CAR-T as most studies to date target tumor-associated antigens, rather than TSAs. Tumor-associated antigens might have variable or heterogeneous expression on tumor cells and may pose a greater risk of off-target toxicity. Serious adverse events have occurred in patients treated with human epidermal growth factor receptor 2–primed^106^ and carcinoembryonic antigen-primed^107^ CAR-T therapy. In addition to tumor antigen selection, tumor-infiltrating lymphocytes and CAR T cells have been shown to become progressively dysfunctional over time and upregulate various inhibitory receptors including PD-1 and lymphocyte-activation gene 3,^108^ making them ineffective to overcome the potently immunosuppressive TME. As with other immunotherapies, adoptive cell transfer alone seems to be inadequate for PDAC treatment, but may play a role in future combination immunotherapy.

### Tumor-Targeted Immunotherapy Strategies

Recently, immunotherapies have been paired with nonimmunologic PDAC-targeted therapies. An ongoing trial combining AB680 (CD73 inhibitor) and zimberelimab (anti–PD-1) with first-line gemcitabine/nab-paclitaxel has demonstrated a tolerable safety profile, with 3 of 9 patients showing PR (one CR) and 5 of 9 with SD.^109^ The SWOG S2001 is an ongoing randomized phase II study (ClinicalTrials.gov identifier: NCT04548752), which seeks to add pembrolizumab to standard-of-care maintenance olaparib for patients with BRCA+ PDAC. It is still to be seen if adding immunotherapies to targeted PDAC regimens will be effective for these select patient populations.

### Future Trial Design

Future trial design is imperative to efficiently gather and accurately assess data to best inform on the complex immune profile of PDAC. Inflammatory-specific end points, such as iRECIST imaging criteria, and correlative studies with explicit aims to evaluate the TME will prove to be invaluable to understand the effects of immune modulation. Paired baseline and on-treatment tumor biopsies have the ability to provide insight into patient-specific responses. These studies should include assessment of the investigational drug's ability to adequately hit its intended target, change the TME, and identify compensatory evasion mechanisms in nonresponders. Proper correlative science will allow for all patients, both responders and nonresponders, to contribute information to the evolving landscape of tumor immunology. With continuing advancement in immune profiling, identification of effective immunotherapies could be on the horizon.

In conclusion, the rising incidence and persistent dismal 5-year OS of PDAC highlight the need for new effective systemic therapies. Immunotherapy has shown significant benefit in solid organ tumors, but has so far been disappointing in the treatment of PDAC. There have been several promising preclinical studies, but translation into the clinic has proved to be challenging. This is likely a result of PDAC's complex TME that protects the tumor against a cytotoxic immune response. The intricate and nonredundant pathways of immune evasion will likely require a combination approach to improve efficacy. Fortunately, many ongoing clinical trials are evaluating combination immunotherapies, which at the minimum, will be able to shed light on mechanisms of immune evasion to educate future trials. It is our belief that through the multidisciplinary approach with engagement of clinicians, scientists, and most importantly patients, immunotherapy will play a key role in the treatment of PDAC in the future.

## References

[1] Rahib L Smith BD Aizenberg R : Projecting cancer incidence and deaths to 2030: The unexpected burden of thyroid, liver, and pancreas cancers in the United States. Cancer Res 74:2913-2921, 2014 24840647 10.1158/0008-5472.CAN-14-0155

[2] Siegel RL Miller KD Fuchs HE : Cancer statistics, 2022. CA Cancer J Clin 72:7-33, 2022 35020204 10.3322/caac.21708

[3] Yoo C Kang J Kim K : Efficacy and safety of neoadjuvant FOLFIRINOX for borderline resectable pancreatic adenocarcinoma: Improved efficacy compared with gemcitabine-based regimen. Oncotarget 8:46337-46347, 2017 28564637 10.18632/oncotarget.17940 PMC5542271

[4] Brahmer JR Drake CG Wollner I : Phase I study of single-agent anti-programmed death-1 (MDX-1106) in refractory solid tumors: Safety, clinical activity, pharmacodynamics, and immunologic correlates. J Clin Oncol 28:3167-3175, 2010 20516446 10.1200/JCO.2009.26.7609 PMC4834717

[5] Topalian SL Sznol M McDermott DF : Survival, durable tumor remission, and long-term safety in patients with advanced melanoma receiving nivolumab. J Clin Oncol 32:1020-1030, 2014 24590637 10.1200/JCO.2013.53.0105 PMC4811023

[6] Ansell SM Lesokhin AM Borrello I : PD-1 blockade with nivolumab in relapsed or refractory Hodgkin's lymphoma. N Engl J Med 372:311-319, 2015 25482239 10.1056/NEJMoa1411087 PMC4348009

[7] Le DT Uram JN Wang H : PD-1 blockade in tumors with mismatch-repair deficiency. N Engl J Med 372:2509-2520, 2015 26028255 10.1056/NEJMoa1500596 PMC4481136

[8] Robert C Long GV Brady B : Nivolumab in previously untreated melanoma without BRAF mutation. N Engl J Med 372:320-330, 2015 25399552 10.1056/NEJMoa1412082

[9] Le DT Durham JN Smith KN : Mismatch repair deficiency predicts response of solid tumors to PD-1 blockade. Science 357:409-413, 2017 28596308 10.1126/science.aan6733 PMC5576142

[10] Royal RE Levy C Turner K : Phase 2 trial of single agent Ipilimumab (anti-CTLA-4) for locally advanced or metastatic pancreatic adenocarcinoma. J Immunother 33:828-833, 2010 20842054 10.1097/CJI.0b013e3181eec14c PMC7322622

[11] Brahmer JR Tykodi SS Chow LQ : Safety and activity of anti-PD-L1 antibody in patients with advanced cancer. N Engl J Med 366:2455-2465, 2012 22658128 10.1056/NEJMoa1200694 PMC3563263

[12] O'Reilly EM Oh DY Dhani N : Durvalumab with or without tremelimumab for patients with metastatic pancreatic ductal adenocarcinoma: A phase 2 randomized clinical trial. JAMA Oncol 5:1431-1438, 2019 31318392 10.1001/jamaoncol.2019.1588 PMC6647002

[13] Yarchoan M Johnson BA III Lutz ER : Targeting neoantigens to augment antitumour immunity. Nat Rev Cancer 17:209-222, 2017 [Erratum: Nat Rev Cancer 17:569, 2017] 28233802 10.1038/nrc.2016.154 PMC5575801

[14] DuPage M Mazumdar C Schmidt LM : Expression of tumour-specific antigens underlies cancer immunoediting. Nature 482:405-409, 2012 22318517 10.1038/nature10803 PMC3288744

[15] Evans RA Diamond MS Rech AJ : Lack of immunoediting in murine pancreatic cancer reversed with neoantigen. JCI Insight 1:e88328, 2016 10.1172/jci.insight.88328 PMC5026128 27642636

[16] Clark CE Hingorani SR Mick R : Dynamics of the immune reaction to pancreatic cancer from inception to invasion. Cancer Res 67:9518-9527, 2007 17909062 10.1158/0008-5472.CAN-07-0175

[17] Vonderheide RH: The immune revolution: A case for priming, not checkpoint. Cancer Cell 33:563-569, 2018 29634944 10.1016/j.ccell.2018.03.008 PMC5898647

[18] Balachandran VP Łuksza M Zhao JN : Identification of unique neoantigen qualities in long-term survivors of pancreatic cancer. Nature 551:512-516, 2017 29132146 10.1038/nature24462 PMC6145146

[19] Bailey P Chang DK Forget MA : Exploiting the neoantigen landscape for immunotherapy of pancreatic ductal adenocarcinoma. Sci Rep 6:35848, 2016 27762323 10.1038/srep35848 PMC5071896

[20] Qian ZR Rubinson DA Nowak JA : Association of alterations in main driver genes with outcomes of patients with resected pancreatic ductal adenocarcinoma. JAMA Oncol 4:e173420, 2018 29098284 10.1001/jamaoncol.2017.3420 PMC5844844

[21] Bayne LJ Beatty GL Jhala N : Tumor-derived granulocyte-macrophage colony-stimulating factor regulates myeloid inflammation and T cell immunity in pancreatic cancer. Cancer Cell 21:822-835, 2012 22698406 10.1016/j.ccr.2012.04.025 PMC3575028

[22] Collins MA Bednar F Zhang Y : Oncogenic Kras is required for both the initiation and maintenance of pancreatic cancer in mice. J Clin Invest 122:639-653, 2012 22232209 10.1172/JCI59227 PMC3266788

[23] Coelho MA de Carné Trécesson S Rana S : Oncogenic RAS signaling promotes tumor immunoresistance by stabilizing PD-L1 mRNA. Immunity 47:1083-1099.e6, 2017 29246442 10.1016/j.immuni.2017.11.016 PMC5746170

[24] Manji GA Wainberg ZA Krishnan K : ARC-8: Phase I/Ib study to evaluate safety and tolerability of AB680 + chemotherapy + zimberelimab (AB122) in patients with treatment-naive metastatic pancreatic adenocarcinoma (mPDAC). J Clin Oncol 39, 2020 (suppl 3; abstr 404)

[25] Dias Carvalho P Guimarães CF Cardoso AP : KRAS oncogenic signaling extends beyond cancer cells to orchestrate the microenvironment. Cancer Res 78:7-14, 2018 29263151 10.1158/0008-5472.CAN-17-2084

[26] McAllister F Bailey JM Alsina J : Oncogenic Kras activates a hematopoietic-to-epithelial IL-17 signaling axis in preinvasive pancreatic neoplasia. Cancer Cell 25:621-637, 2014 24823639 10.1016/j.ccr.2014.03.014 PMC4072043

[27] Yamamoto K Venida A Yano J : Autophagy promotes immune evasion of pancreatic cancer by degrading MHC-I. Nature 581:100-105, 2020 32376951 10.1038/s41586-020-2229-5 PMC7296553

[28] Yang S Wang X Contino G : Pancreatic cancers require autophagy for tumor growth. Genes Dev 25:717-729, 2011 21406549 10.1101/gad.2016111 PMC3070934

[29] McCracken MN Cha AC Weissman IL: Molecular pathways: Activating T cells after cancer cell phagocytosis from blockade of CD47 “don't eat me” signals. Clin Cancer Res 21:3597-3601, 2015 26116271 10.1158/1078-0432.CCR-14-2520 PMC4621226

[30] Tseng D Volkmer JP Willingham SB : Anti-CD47 antibody-mediated phagocytosis of cancer by macrophages primes an effective antitumor T-cell response. Proc Natl Acad Sci USA 110:11103-11108, 2013 23690610 10.1073/pnas.1305569110 PMC3703977

[31] Uyttenhove C Pilotte L Théate I : Evidence for a tumoral immune resistance mechanism based on tryptophan degradation by indoleamine 2,3-dioxygenase. Nat Med 9:1269-1274, 2003 14502282 10.1038/nm934

[32] Jang JE Hajdu CH Liot C : Crosstalk between regulatory T cells and tumor-associated dendritic cells negates anti-tumor immunity in pancreatic cancer. Cell Rep 20:558-571, 2017 28723561 10.1016/j.celrep.2017.06.062 PMC5649374

[33] Hanahan D Coussens LM: Accessories to the crime: Functions of cells recruited to the tumor microenvironment. Cancer Cell 21:309-322, 2012 22439926 10.1016/j.ccr.2012.02.022

[34] Erkan M Michalski CW Rieder S : The activated stroma index is a novel and independent prognostic marker in pancreatic ductal adenocarcinoma. Clin Gastroenterol Hepatol 6:1155-1161, 2008 18639493 10.1016/j.cgh.2008.05.006

[35] Wang LM Silva MA D'Costa Z : The prognostic role of desmoplastic stroma in pancreatic ductal adenocarcinoma. Oncotarget 7:4183-4194, 2016 26716653 10.18632/oncotarget.6770 PMC4826198

[36] Provenzano PP Cuevas C Chang AE : Enzymatic targeting of the stroma ablates physical barriers to treatment of pancreatic ductal adenocarcinoma. Cancer Cell 21:418-429, 2012 22439937 10.1016/j.ccr.2012.01.007 PMC3371414

[37] Maj T Wang W Crespo J : Oxidative stress controls regulatory T cell apoptosis and suppressor activity and PD-L1-blockade resistance in tumor. Nat Immunol 18:1332-1341, 2017 29083399 10.1038/ni.3868 PMC5770150

[38] Jacobetz MA Chan DS Neesse A : Hyaluronan impairs vascular function and drug delivery in a mouse model of pancreatic cancer. Gut 62:112-120, 2013 22466618 10.1136/gutjnl-2012-302529 PMC3551211

[39] Ene-Obong A Clear AJ Watt J : Activated pancreatic stellate cells sequester CD8+ T cells to reduce their infiltration of the juxtatumoral compartment of pancreatic ductal adenocarcinoma. Gastroenterology 145:1121-1132, 2013 23891972 10.1053/j.gastro.2013.07.025 PMC3896919

[40] Jiang H Hegde S Knolhoff BL : Targeting focal adhesion kinase renders pancreatic cancers responsive to checkpoint immunotherapy. Nat Med 22:851-860, 2016 27376576 10.1038/nm.4123 PMC4935930

[41] Feig C Jones JO Kraman M : Targeting CXCL12 from FAP-expressing carcinoma-associated fibroblasts synergizes with anti-PD-L1 immunotherapy in pancreatic cancer. Proc Natl Acad Sci USA 110:20212-20217, 2013 24277834 10.1073/pnas.1320318110 PMC3864274

[42] Mace TA Bloomston M Lesinski GB: Pancreatic cancer-associated stellate cells: A viable target for reducing immunosuppression in the tumor microenvironment. Oncoimmunology 2:e24891, 2013 24073373 10.4161/onci.24891 PMC3782129

[43] Waghray M Yalamanchili M Dziubinski M : GM-CSF mediates mesenchymal-epithelial cross-talk in pancreatic cancer. Cancer Discov 6:886-899, 2016 27184426 10.1158/2159-8290.CD-15-0947 PMC5549011

[44] Jiang H Hegde S DeNardo DG: Tumor-associated fibrosis as a regulator of tumor immunity and response to immunotherapy. Cancer Immunol Immunother 66:1037-1048, 2017 28451791 10.1007/s00262-017-2003-1 PMC5603233

[45] Mitchem JB Brennan DJ Knolhoff BL : Targeting tumor-infiltrating macrophages decreases tumor-initiating cells, relieves immunosuppression, and improves chemotherapeutic responses. Cancer Res 73:1128-1141, 2013 23221383 10.1158/0008-5472.CAN-12-2731 PMC3563931

[46] Gabrilovich D Nagaraj S: Myeloid-derived suppressor cells as regulators of the immune system. Nat Rev Immunol 9:162-174, 2009 19197294 10.1038/nri2506 PMC2828349

[47] Kumar V Patel S Tcyganov E : The nature of myeloid-derived suppressor cells in the tumor microenvironment. Trends Immunol 37:208-220, 2016 26858199 10.1016/j.it.2016.01.004 PMC4775398

[48] Balachandran VP Beatty GL Dougan SK: Broadening the impact of immunotherapy to pancreatic cancer: Challenges and opportunities. Gastroenterology 156:2056-2072.64, 2019 30660727 10.1053/j.gastro.2018.12.038 PMC6486864

[49] Zhu Y Herndon JM Sojka DK : Tissue-resident macrophages in pancreatic ductal adenocarcinoma originate from embryonic hematopoiesis and promote tumor progression. Immunity 47:323-338.e6, 2017 [Erratum: Immunity 47:597, 2017] 28813661 10.1016/j.immuni.2017.07.014 PMC5578409

[50] Sanford DE Belt BA Panni RZ : Inflammatory monocyte mobilization decreases patient survival in pancreatic cancer: A role for targeting the CCL2/CCR2 axis. Clin Cancer Res 19:3404-3415, 2013 23653148 10.1158/1078-0432.CCR-13-0525 PMC3700620

[51] Gabrilovich DI Ostrand-Rosenberg S Bronte V: Coordinated regulation of myeloid cells by tumours. Nat Rev Immunol 12:253-268, 2012 22437938 10.1038/nri3175 PMC3587148

[52] Steele CW Karim SA Leach JDG : CXCR2 inhibition profoundly suppresses metastases and augments immunotherapy in pancreatic ductal adenocarcinoma. Cancer Cell 29:832-845, 2016 27265504 10.1016/j.ccell.2016.04.014 PMC4912354

[53] Ruffell B Chang-Strachan D Chan V : Macrophage IL-10 blocks CD8+ T cell-dependent responses to chemotherapy by suppressing IL-12 expression in intratumoral dendritic cells. Cancer Cell 26:623-637, 2014 25446896 10.1016/j.ccell.2014.09.006 PMC4254570

[54] Zhang Y Velez-Delgado A Mathew E : Myeloid cells are required for PD-1/PD-L1 checkpoint activation and the establishment of an immunosuppressive environment in pancreatic cancer. Gut 66:124-136, 2017 27402485 10.1136/gutjnl-2016-312078 PMC5256390

[55] Daley D Mani VR Mohan N : Dectin 1 activation on macrophages by galectin 9 promotes pancreatic carcinoma and peritumoral immune tolerance. Nat Med 23:556-567, 2017 28394331 10.1038/nm.4314 PMC5419876

[56] Chao T Furth EE Vonderheide RH: CXCR2-Dependent accumulation of tumor-associated neutrophils regulates T-cell immunity in pancreatic ductal adenocarcinoma. Cancer Immunol Res 4:968-982, 2016 27737879 10.1158/2326-6066.CIR-16-0188 PMC5110270

[57] Stromnes IM Brockenbrough JS Izeradjene K : Targeted depletion of an MDSC subset unmasks pancreatic ductal adenocarcinoma to adaptive immunity. Gut 63:1769-1781, 2014 24555999 10.1136/gutjnl-2013-306271 PMC4340484

[58] Nywening TM Belt BA Cullinan DR : Targeting both tumour-associated CXCR2+ neutrophils and CCR2+ macrophages disrupts myeloid recruitment and improves chemotherapeutic responses in pancreatic ductal adenocarcinoma. Gut 67:1112-1123, 2018 29196437 10.1136/gutjnl-2017-313738 PMC5969359

[59] Panni RZ Herndon JM Zuo C : Agonism of CD11b reprograms innate immunity to sensitize pancreatic cancer to immunotherapies. Sci Transl Med 11:eaau9240, 2019 31270275 10.1126/scitranslmed.aau9240 PMC7197026

[60] Lin JH Huffman AP Wattenberg MM : Type 1 conventional dendritic cells are systemically dysregulated early in pancreatic carcinogenesis. J Exp Med 217:e20190673, 2020 32453421 10.1084/jem.20190673 PMC7398166

[61] Bottcher JP Reis e Sousa C: The role of type 1 conventionaldendritic cells in cancer immunity. Trends Cancer 4:784-792, 2018 30352680 10.1016/j.trecan.2018.09.001 PMC6207145

[62] Gunderson AJ Kaneda MM Tsujikawa T : Bruton tyrosine kinase-dependent immune cell cross-talk drives pancreas cancer. Cancer Discov 6:270-285, 2016 [Erratum: Cancer Discov 6:802, 2019] 26715645 10.1158/2159-8290.CD-15-0827 PMC4783268

[63] Lee KE Spata M Bayne LJ : Hif1a deletion reveals pro-neoplastic function of B cells in pancreatic neoplasia. Cancer Discov 6:256-269, 2016 26715642 10.1158/2159-8290.CD-15-0822 PMC4783189

[64] Stromnes IM Hulbert A Pierce RH : T-cell localization, activation, and clonal expansion in human pancreatic ductal adenocarcinoma. Cancer Immunol Res 5:978-991, 2017 29066497 10.1158/2326-6066.CIR-16-0322 PMC5802342

[65] Tassi E Gavazzi F Albarello L : Carcinoembryonic antigen-specific but not antiviral CD4+ T cell immunity is impaired in pancreatic carcinoma patients. J Immunol 181:6595-6603, 2008 18941250 10.4049/jimmunol.181.9.6595

[66] Ino Y Yamazaki-Itoh R Shimada K : Immune cell infiltration as an indicator of the immune microenvironment of pancreatic cancer. Br J Cancer 108:914-923, 2013 23385730 10.1038/bjc.2013.32 PMC3590668

[67] Hiraoka N Onozato K Kosuge T : Prevalence of FOXP3+ regulatory T cells increases during the progression of pancreatic ductal adenocarcinoma and its premalignant lesions. Clin Cancer Res 12:5423-5434, 2006 17000676 10.1158/1078-0432.CCR-06-0369

[68] Tan MC Goedegebuure PS Belt BA : Disruption of CCR5-dependent homing of regulatory T cells inhibits tumor growth in a murine model of pancreatic cancer. J Immunol 182:1746-1755, 2009 19155524 10.4049/jimmunol.182.3.1746 PMC3738070

[69] Linehan JD Kolios G Valatas V : Immunomodulatory cytokines suppress epithelial nitric oxide production in inflammatory bowel disease by acting on mononuclear cells. Free Radic Biol Med 39:1560-1569, 2005 16298681 10.1016/j.freeradbiomed.2005.07.019

[70] Poschke I Faryna M Bergmann F : Identification of a tumor-reactive T-cell repertoire in the immune infiltrate of patients with resectable pancreatic ductal adenocarcinoma. Oncoimmunology 5:e1240859, 2016 28123878 10.1080/2162402X.2016.1240859 PMC5215250

[71] Fan JQ Wang MF Chen HL : Current advances and outlooks in immunotherapy for pancreatic ductal adenocarcinoma. Mol Cancer 19:32, 2020 32061257 10.1186/s12943-020-01151-3 PMC7023714

[72] Bengsch F Knoblock DM Liu A : CTLA-4/CD80 pathway regulates T cell infiltration into pancreatic cancer. Cancer Immunol Immunother 66:1609-1617, 2017 28856392 10.1007/s00262-017-2053-4 PMC5677559

[73] Van Cutsem E Tempero MA Sigal D : Randomized phase III trial of Pegvorhyaluronidase alfa with nab-paclitaxel plus gemcitabine for patients with hyaluronan-high metastatic pancreatic adenocarcinoma. J Clin Oncol 38:3185-3194, 2020 32706635 10.1200/JCO.20.00590 PMC7499614

[74] Bockorny B Semenisty V Macarulla T : BL-8040, a CXCR4 antagonist, in combination with pembrolizumab and chemotherapy for pancreatic cancer: The COMBAT trial. Nat Med 26:878-885, 2020 32451495 10.1038/s41591-020-0880-x

[75] O'Hara MH Messersmith W Kindler H : Safety and pharmacokinetics of CXCR4 peptide antagonist, LY2510924, in combination with durvalumab in advanced refractory solid tumors. J Pancreat Cancer 6:21-31, 2020 32219196 10.1089/pancan.2019.0018 PMC7097682

[76] Wang-Gillam A McWilliams R Lockhart AC : Phase I study of defactinib combined with pembrolizumab and gemcitabine in patients with advanced cancer: Experiences of pancreatic ductal adenocarcinoma (PDAC) patients. Cancer Res 80:CT118, 2020

[77] Komar HM Serpa G Kerscher C : Inhibition of Jak/STAT signaling reduces the activation of pancreatic stellate cells in vitro and limits caerulein-induced chronic pancreatitis in vivo. Sci Rep 7:1787, 2017 28496202 10.1038/s41598-017-01973-0 PMC5431930

[78] Hurwitz H Van Cutsem E Bendell J : Ruxolitinib + capecitabine in advanced/metastatic pancreatic cancer after disease progression/intolerance to first-line therapy: JANUS 1 and 2 randomized phase III studies. Invest New Drugs 36:683-695, 2018 29508247 10.1007/s10637-018-0580-2 PMC6752723

[79] Cho BC Nathan B Bendell J : Phase Ib/II open-label, randomized evaluation of atezolizumab + cobimetinib vs control in MORPHEUS-NSCLC (non-small cell lung cancer), MORPHEUS-PDAC (pancreatic ductal adenocarcinoma) and MORPHEUS-GC (gastric cancer). Cancer Res 80, 2020 (suppl 16; abstr CT201)

[80] Nywening TM Wang-Gillam A Sanford DE : Targeting tumour-associated macrophages with CCR2 inhibition in combination with FOLFIRINOX in patients with borderline resectable and locally advanced pancreatic cancer: A single-centre, open-label, dose-finding, non-randomised, phase 1b trial. Lancet Oncol 17:651-662, 2016 27055731 10.1016/S1470-2045(16)00078-4 PMC5407285

[81] Noel M O'Reilly EM Wolpin BM : Phase 1b study of a small molecule antagonist of human chemokine (C-C motif) receptor 2 (PF-04136309) in combination with nab-paclitaxel/gemcitabine in first-line treatment of metastatic pancreatic ductal adenocarcinoma. Invest New Drugs 38:800-811, 2020 31297636 10.1007/s10637-019-00830-3 PMC7211198

[82] Zhu Y Knolhoff BL Meyer MA : CSF1/CSF1R blockade reprograms tumor-infiltrating macrophages and improves response to T-cell checkpoint immunotherapy in pancreatic cancer models. Cancer Res 74:5057-5069, 2014 25082815 10.1158/0008-5472.CAN-13-3723 PMC4182950

[83] Rasco DW Bendell JC Wang-Gillam A : A phase I/II study of GB1275, a first-in-class oral CD11b modulator, alone, and combined with pembrolizumab in specified advanced solid tumors or with chemotherapy in metastatic pancreatic cancer (KEYNOTE-A36). J Clin Oncol 38, 2020 (suppl 15; abstr 3085)

[84] Massó-Vallés D Jauset T Serrano E : Ibrutinib exerts potent antifibrotic and antitumor activities in mouse models of pancreatic adenocarcinoma. Cancer Res 75:1675-1681, 2015 25878147 10.1158/0008-5472.CAN-14-2852 PMC6773609

[85] Tempero M Oh DY Tabernero J : Ibrutinib in combination with nab-paclitaxel and gemcitabine for first-line treatment of patients with metastatic pancreatic adenocarcinoma: Phase III RESOLVE study. Ann Oncol 32:600-608, 2021 33539945 10.1016/j.annonc.2021.01.070

[86] Beatty GL Li Y Long KB: Cancer immunotherapy: Activating innate and adaptive immunity through CD40 agonists. Expert Rev Anticancer Ther 17:175-186, 2017 27927088 10.1080/14737140.2017.1270208 PMC5533512

[87] Byrne KT Betts CB Mick R : Neoadjuvant selicrelumab, an agonist CD40 antibody, induces changes in the tumor microenvironment in patients with resectable pancreatic cancer. Clin Cancer Res 27:4574-4586, 2021 34112709 10.1158/1078-0432.CCR-21-1047 PMC8667686

[88] Beatty GL Torigian DA Chiorean EG : A phase I study of an agonist CD40 monoclonal antibody (CP-870,893) in combination with gemcitabine in patients with advanced pancreatic ductal adenocarcinoma. Clin Cancer Res 19:6286-6295, 2013 23983255 10.1158/1078-0432.CCR-13-1320 PMC3834036

[89] Zippelius A Schreiner J Herzig P : Induced PD-L1 expression mediates acquired resistance to agonistic anti-CD40 treatment. Cancer Immunol Res 3:236-244, 2015 25623164 10.1158/2326-6066.CIR-14-0226

[90] O'Hara MH O'Reilly EM Varadhachary G : CD40 agonistic monoclonal antibody APX005M (sotigalimab) and chemotherapy, with or without nivolumab, for the treatment of metastatic pancreatic adenocarcinoma: An open-label, multicentre, phase 1b study. Lancet Oncol 22:118-131, 2021 33387490 10.1016/S1470-2045(20)30532-5

[91] O'Hara MH O'Reilly EM Wolff RA : Gemcitabine (Gem) and nab-paclitaxel (NP) ± nivolumab (nivo) ± CD40 agonistic monoclonal antibody APX005M (sotigalimab), in patients (Pts) with untreated metastatic pancreatic adenocarcinoma (mPDAC): Phase (Ph) 2 final results. J Clin Oncol 39, 2021 (suppl 15; abstr 4019) 10.1016/S1470-2045(20)30532-5 33387490

[92] Lutz ER Wu AA Bigelow E : Immunotherapy converts nonimmunogenic pancreatic tumors into immunogenic foci of immune regulation. Cancer Immunol Res 2:616-631, 2014 24942756 10.1158/2326-6066.CIR-14-0027 PMC4082460

[93] Le DT Wang-Gillam A Picozzi V : Safety and survival with GVAX pancreas prime and Listeria Monocytogenes-expressing mesothelin (CRS-207) boost vaccines for metastatic pancreatic cancer. J Clin Oncol 33:1325-1333, 2015 25584002 10.1200/JCO.2014.57.4244 PMC4397277

[94] Le DT Picozzi VJ Ko AH : Results from a phase IIb, randomized, multicenter study of GVAX pancreas and CRS-207 compared with chemotherapy in adults with previously treated metastatic pancreatic adenocarcinoma (ECLIPSE study). Clin Cancer Res 25:5493-5502, 2019 31126960 10.1158/1078-0432.CCR-18-2992 PMC7376746

[95] Le DT Crocenzi TS Uram JN : Randomized phase II study of the safety, efficacy, and immune response of GVAX pancreas vaccine (with cyclophosphamide) and CRS-207 with or without nivolumab in patients with previously treated metastatic pancreatic adenocarcinoma (STELLAR). J Clin Oncol 33, 2015(suppl 15; abstr TPS4148)

[96] Hardacre JM Mulcahy M Small W : Addition of algenpantucel-L immunotherapy to standard adjuvant therapy for pancreatic cancer: A phase 2 study. J Gastrointest Surg 17:94-100, 2013; discussion 100-101 23229886 10.1007/s11605-012-2064-6

[97] Hewitt DB Nissen N Hatoum H : A phase 3 randomized clinical trial of chemotherapy with or without algenpantucel-L (HyperAcute-Pancreas) immunotherapy in subjects with borderline resectable or locally advanced unresectable pancreatic cancer. Ann Surg 275:45-53, 2022 33630475 10.1097/SLA.0000000000004669

[98] Behrens ME Grandgenett PM Bailey JM : The reactive tumor microenvironment: MUC1 signaling directly reprograms transcription of CTGF. Oncogene 29:5667-5677, 2010 20697347 10.1038/onc.2010.327 PMC3412169

[99] Ramanathan RK Lee KM McKolanis J : Phase I study of a MUC1 vaccine composed of different doses of MUC1 peptide with SB-AS2 adjuvant in resected and locally advanced pancreatic cancer. Cancer Immunol Immunother 54:254-264, 2005 15372205 10.1007/s00262-004-0581-1 PMC11034344

[100] Abou-Alfa GK Chapman PB Feilchenfeldt J : Targeting mutated K-ras in pancreatic adenocarcinoma using an adjuvant vaccine. Am J Clin Oncol 34:321-325, 2011 20686403 10.1097/COC.0b013e3181e84b1f

[101] Wedén S Klemp M Gladhaug IP : Long-term follow-up of patients with resected pancreatic cancer following vaccination against mutant K-ras. Int J Cancer 128:1120-1128, 2011 20473937 10.1002/ijc.25449

[102] June CH: Adoptive T cell therapy for cancer in the clinic. J Clin Invest 117:1466-1476, 2007 17549249 10.1172/JCI32446 PMC1878537

[103] Kondo H Hazama S Kawaoka T : Adoptive immunotherapy for pancreatic cancer using MUC1 peptide-pulsed dendritic cells and activated T lymphocytes. Anticancer Res 28:379-387, 2008 18383873

[104] Matsui H Hazama S Sakamoto K : Postoperative adjuvant therapy for resectable pancreatic cancer with gemcitabine and adoptive immunotherapy. Pancreas 46:994-1002, 2017 28697053 10.1097/MPA.0000000000000880 PMC5555975

[105] Smaglo BG Musher BL Vasileiou S : A phase I trial targeting advanced or metastatic pancreatic cancer using a combination of standard chemotherapy and adoptively transferred nonengineered, multiantigen specific T cells in the first-line setting (TACTOPS). J Clin Oncol 38, 2020 (suppl 15; abstr 4622)

[106] Morgan RA Yang JC Kitano M : Case report of a serious adverse event following the administration of T cells transduced with a chimeric antigen receptor recognizing ERBB2. Mol Ther 18:843-851, 2010 20179677 10.1038/mt.2010.24 PMC2862534

[107] Thistlethwaite FC Gilham DE Guest RD : The clinical efficacy of first-generation carcinoembryonic antigen (CEACAM5)-specific CAR T cells is limited by poor persistence and transient pre-conditioning-dependent respiratory toxicity. Cancer Immunol Immunother 66:1425-1436, 2017 28660319 10.1007/s00262-017-2034-7 PMC5645435

[108] Stromnes IM Schmitt TM Hulbert A : T cells engineered against a native antigen can surmount immunologic and physical barriers to treat pancreatic ductal adenocarcinoma. Cancer Cell 28:638-652, 2015 26525103 10.1016/j.ccell.2015.09.022 PMC4724422

[109] Manji GA Wainberg ZA Krishnan K : ARC-8: Phase I/Ib study to evaluate safety and tolerability of AB680 + chemotherapy + zimberelimab (AB122) in patients with treatment-naive metastatic pancreatic adenocarcinoma (mPDAC). J Clin Oncol 39, 2021 (suppl 3; abstr 404)

